# Nebulised *N*-Acetylcysteine for Unresponsive Bronchial Obstruction in Allergic Brochopulmonary Aspergillosis: A Case Series and Review of the Literature

**DOI:** 10.3390/jof4040117

**Published:** 2018-10-15

**Authors:** Akaninyene Otu, Philip Langridge, David W. Denning

**Affiliations:** 1 Department of Internal Medicine, College of Medical Sciences, University of Calabar, Calabar, Cross River State P.M.B. 1115, Nigeria; 2 The National Aspergillosis Centre, 2nd Floor Education and Research Centre, Wythenshawe Hospital, Southmoor Road, Manchester M23 9LT, UK; philip.langridge@mft.nhs.uk (P.L.); ddenning@manchester.ac.uk (D.W.D.); 3 Faculty of Biology, Medicine and Health, The University of Manchester, and Manchester Academic Health Science Centre, Oxford Rd, Manchester M13 9PL, UK

**Keywords:** *N*-acetylcysteine, mucolytic, allergic bronchopulmonary aspergillosis

## Abstract

Many chronic lung diseases are characterized by the hypersecretion of mucus. In these conditions, the administration of mucoactive agents is often indicated as adjuvant therapy. *N*-acetylcysteine (NAC) is a typical example of a mucolytic agent. A retrospective review of patients with pulmonary aspergillosis treated at the National Aspergillosis Centre in Manchester, United Kingdom, with NAC between November 2015 and November 2017 was carried out. Six Caucasians with *Aspergillus* lung disease received NAC to facilitate clearance of their viscid bronchial mucus secretions. One patient developed immediate bronchospasm on the first dose and could not be treated. Of the remainder, two (33%) derived benefit, with increased expectoration and reduced symptoms. Continued response was sustained over 6–7 months, without any apparent toxicity. In addition, a systematic review of the literature is provided to analyze the utility of NAC in the management of respiratory conditions which have unresponsive bronchial obstruction as a feature.

## 1. Introduction

Despite constant exposure to pathogens, particles and toxic chemicals, the lungs have intrinsic mechanisms which prevent environmental injury. The airway mucociliary clearance apparatus is vital for preventing lung disease in humans. This apparatus clears mucus by ciliary movement and sputum is cleared by cough. The integrity of this apparatus is determined by several factors, namely the ciliary function, the property and volume of mucus and the mucociliary interactions [1]. Mucus typically consists of water (97%) and mucins (3%), such as MUC5AC and MUC5B, in addition to antimicrobial, immunomodulatory and protective molecules. Mucus prevents dehydration on the airway surface and aids in clearance of inhaled particles and inflammatory mediators [2]. Many chronic lung diseases are characterized by the hypersecretion of mucus which arises from hypertrophy and hyperplasia of the goblet and submucosal glands. This is usually accompanied by inadequate mucus clearance which further hinders the air passages. In these conditions, the administration of mucoactive agents is often indicated as adjuvant therapy. Mucoactive substances act to increase the ability to expectorate sputum or to decrease mucus hypersecretion [2]. Expectorants and mucolytics are examples of mucoactive substances. *N*-acetylcysteine (NAC) is a mucolytic.

Six patients with *Aspergillus* lung disease, who received NAC to facilitate clearance of their viscid bronchial mucus secretions, were reported. In addition, we conducted a systematic review of the literature to analyze the utility of NAC in the management of respiratory conditions which have unresponsive bronchial obstruction as a feature.

## 2. Methods

We carried out a retrospective review of patients with aspergillosis treated at the National Aspergillosis Centre in Manchester, United Kingdom, with NAC between November 2015 and November 2017.

The National Aspergillosis Centre treats people with a variety of manifestations of aspergillosis. Respiratory physiotherapists were initially recruited to the National Aspergillosis Centre team to procure these sputum samples during clinic visits and this role has evolved over time. In this center, the physiotherapists facilitate the provision of NAC to patients with pulmonary aspergillosis who produce viscid, or inspissated mucous secretions. Here, the NAC treatment is reserved for those symptomatic patients who have tried routine interventions such as physiotherapy, routine suction, nebulization of hypertonic saline with no improvement. As inhalation of NAC may elicit bronchospasm, a challenge test is usually conducted by a senior physiotherapist with spirometry pre, during and post the initial dose. The challenge test is usually carried out in an isolated room to limit the exposure of other persons to NAC. As NAC is denatured by oxygen, nebulizers are usually air-driven. The nebulizer of choice is the Pari Sprint to enhance airway deposition. A typical dose would be 4mL of a 20% solution of NAC (Mucomyst^®^). In cases of suspected or confirmed sensitivity to the 20% solution, the lower dosage is used. After proper administration of NAC, an increased volume of liquefied bronchial secretions may occur. The airway is maintained by cough, physiotherapy or mechanical suction as necessary. Most patients with NAC-related bronchospasm are quickly relieved by the use of bronchodilator given by nebulization. A >15% reduction in FEV_1_ caused by nebulization of NAC is regarded as an indication for “rescue” bronchodilator.

## 3. Results

The data from six Caucasians is summarized in Table 1. There were four males and two females with an average age of 59.8 years. The diagnosis of these patients ranged from allergic bronchopulmonary aspergillosis (ABPA) [four patients] to aspergillus bronchitis (one patient) in addition to other non-infectious co-morbidities. One of the patients had ABPA complicated by chronic pulmonary aspergillosis (CPA).

Over the last 2 years, a total of six patients with aspergillosis have been treated with NAC to facilitate clearance of their viscid bronchial mucus secretions. Details of these patients and the outcome of NAC therapy are provided in Table 1. All patients had failed other treatments aimed at clearing their mucus. One patient developed immediate bronchospasm on the first dose and could not be treated. Of the remainder, two (33%) derived benefit, with increased expectoration and reduced symptoms. Continued response was sustained over 6–7 months, without any apparent toxicity.

## 4. Literature Review

Expectorants increase airway water or the volume of airway secretions, thereby improving the ability to expectorate purulent secretions [3]. Hypertonic saline is an example of an expectorant which has osmotic pressure greater than that of physiologic isotonic 0.9% NaCl. Hypertonic saline has been shown to significantly reduce the number of exacerbations in cystic fibrosis patients when compared to isotonic saline [4] and has better mucociliary clearance [5]. Mucolytics act by decreasing the viscosity of secretions which they achieve by degrading fibrin, DNA and mucin polymers or F-actin [3]. There are three basic classes of mucolytics, namely the peptide mucolytics, the nondestructive mucolytics and the classical mucolytics. The peptide mucolytics are found to be effective when sputum contains a lot of DNA pus and acts to depolymerize the DNA polymer (dornase alfa) or the F-actin network. Dornase alfa is an example of a peptide mucolytic which is used to treat patients with cystic fibrosis [6]. The nondestructive mucolytics act to untangle the charged oligosaccharide side chains of mucin and examples of this are heparin and low molecular weight dextran [3]. Classic mucolytics hydrolyze the disulfide bonds that link the mucin monomers and NAC, also known as acetylcysteine or *N*-acetyl-l-cysteine, are an example of a classic mucolytic. Details of the various mucoactive substances and their clinical uses are provided in Table 2.

### 4.1. N-Acetylcysteine—The Archetypal Classical Mucolytic

**Figure 1:**
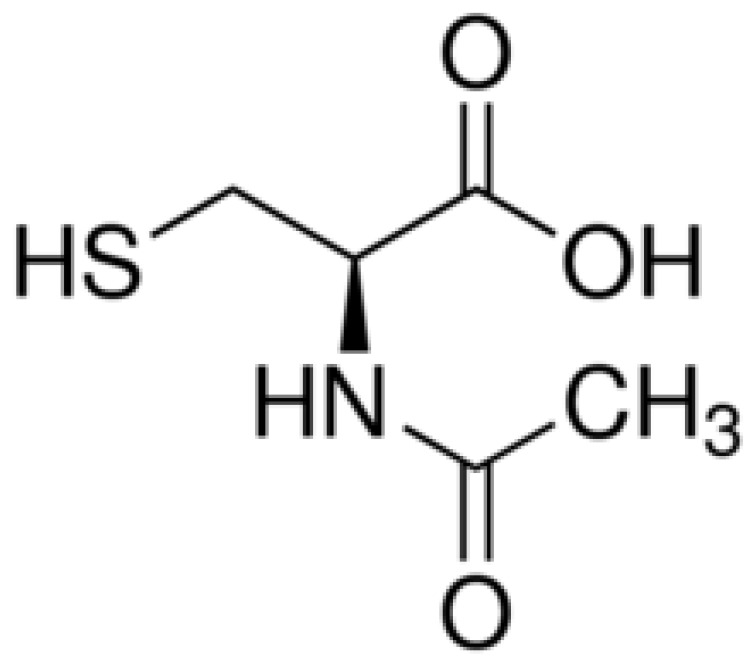
Chemical structure of NAC.

NAC is a synthetic derivative of the endogenous amino acid L-cysteine which is a precursor of the biologic antioxidant glutathione. NAC has been used as a mucolytic for five decades. It has the chemical formula C_5_H_9_NO_3_S with a molecular weight of 163.2 g/mol. NAC is comprised of propanoic acid, in which the carboxyl group is substituted at R_3_ with a sulfanyl group, and at R_2_ with an acetamide group (Figure 1). The antioxidant effects of NAC have been attributed to the thiol (sulfhydryl) group which causes it to be able to reduce free radicals.

There are several modes of administration of NAC and these include nasal instillation, nebulizer, bronchoscopic instillation and intravenous (IV) NAC. IV NAC has been used as a very effective therapy for paracetamol (acetaminophen) poisoning. Early administration of NAC after suspected paracetamol overdose is essential [18] as it is nearly 100% hepatoprotective when it is given within 8–10 h post ingestion [18,19].

NAC has anti-inflammatory and antioxidant properties and has been used in patients with chronic obstructive pulmonary disease (COPD) and rhinosinusitis [1] with variable results. The antioxidant effects of NAC are thought to be mediated by increased levels of intracellular reduced glutathione in the lungs and neutralizing oxidant species [20]. Its mucolytic action is attributed to its effect of reducing sulfhydryl moieties, thereby leading to a disruption of disulfide bridges within the glycoprotein matrix of mucus [20]. This causes a reduction in the viscoelasticity of mucus and brings the levels closer to the optimal level, thereby making it easier to transport along the airways.

There is some evidence that NAC has antimicrobial and anti-biofilm properties [21]. As NAC is commonly administered with antibiotics in the setting of chronic lung disease, its potential modulatory effect on antibiotic activity has been studied extensively. Synergism with ampicillin and antagonism with macrolides, fluoroquinolones and aminoglycosides have been reported [22]. However, other researchers have attributed this apparent synergism to the low pH created by pure NAC powder solutions and not to the NAC itself [23]. Following an investigation into the effect of high NAC concentrations (10 and 50 mM) on antibiotic activity against 40 strains of respiratory pathogens, Landini and colleagues reported a dose-dependent reduction in the activity of carbapenems. A concentration of 50 mM NAC was shown to sporadically decrease ceftriaxone and aminoglycoside antibiotic activity while increasing that of penicillins [24].

There are several reports of a reduction in the frequency of exacerbations and improvement in FEV1 among COPD patients [25,26] with administration of NAC, but other researchers have found no benefits accruing from its use [27,28]. Three recent meta-analyses have explored the effect of NAC on COPD assessing the total number of exacerbations, the number of patients with one exacerbation and FEV1 as outcomes. One analysis found a reduction of exacerbations with both low- and high-dose NAC [29], while the other suggested improvements only with high-dose NAC [30]. The most recent analysis reports that high-dose and low-dose NAC reduced COPD exacerbation frequency. Long-term but not short-term NAC reduced exacerbation prevalence but did not affect lung volumes, namely FEV_1_, forced vital capacity (FVC), or inspiratory capacity (IC) [31].

With respect to use of NAC in patients with cystic fibrosis, a recent Cochrane review found that both nebulized and oral NAC were well tolerated with no major adverse events. However, this review found no evidence to recommend the use of either nebulized or oral NAC in people with cystic fibrosis [32]. The findings of a meta-analysis to assess the possible prophylactic benefit of oral NAC in chronic bronchitis suggest that prolonged NAC prevents acute exacerbations of chronic bronchitis, thus possibly decreasing morbidity and health care costs [33].

### 4.2. Pharmacokinetics of N-Acetylcysteine

NAC can exist in plasma as the parent compound, *N*-acetylcysteine, *N*,*N*-diacetylcysteine and cysteine. Following an oral administration of 200 mg to 600 mg of NAC, it gets rapidly absorbed and achieves peak plasma concentrations after about 0.5 h to 1 h [34]. NAC has a low oral bioavailability of 4–10%, and this may be related to metabolism that occurs within the gut wall and first pass metabolism within the liver [35,36]. About 30% of clearance occurs renally [36]. The terminal half-life of total acetylcysteine after oral doses is 6.25 h while the terminal half-life for reduced and total acetylcysteine following an IV dosage are 1.95 and 5.58 h, respectively [35].

### 4.3. N-Acetylcysteine Products on the Market

There are several products containing NAC on the market today. These products range from sterile solutions (not for injection) to solutions for IV administration, capsules, effervescent tablets, granules and dry syrups. Details of these products are shown in Table 3.

### 4.4. Nebulized versus Oral N-Acetylcysteine

Nebulized NAC appears to have a different mechanism of action from oral NAC. The nebulized NAC is delivered directly to the lower airway and opens up disulfide bonds in mucoproteins, thereby lowering the viscosity of pulmonary secretions. Research has shown that there is no detectable NAC in bronchoalveolar lavage following oral administration [37]. This suggests that oral NAC is unlikely to have any mucolytic properties. A controlled, double-blind, crossover study investigating the mucolytic effects of regular oral NAC in nine patients with chronic bronchitis showed no significant differences in lung function, mucociliary clearance curves or sputum viscosity compared to control or placebo measurements [38].

When administered orally, NAC is deacetylated to cysteine, which is comprised of a thiol group that has reducing and antioxidant properties [39]. The sulfhydryl group in NAC is thought to mediate a reduction in pulmonary oxidative stress and inflammation [40].

### 4.5. Asthma with Hypersecretion of Mucus—Use of N-Acetylcysteine Down A Bronchoscope

Increased mucus secretions with formation of mucus plugs within the airways is one of the three major characteristics of asthma. Asthma may present with a spectrum ranging from mild to fatal disease and the mortality associated with acute severe asthma ranges from 1% to 10% [41]. The life-threatening form of asthma sometimes requires mechanical ventilation. The role of bronchoscopy in this setting is not well established [42]. However, there are several case reports supporting the efficacy of NAC administered through a bronchoscope for patients with status asthmaticus. In a patient with status asthmaticus complicated by mucus impaction, pulmonary lavage was done twice in 24 h using 30 mL of 20% NAC, 250 mL normal saline, 0.5 mL Bronkosol and 125 mg Solu-Medrol. After this procedure, a marked improvement was recorded and extubation was accomplished within 48 h [43]. A case series reports significant results in three patients with severe chronic bronchial asthma who underwent bronchoscopy and lavage, using NAC, Solu-Medrol and isoetharine in the irrigation fluid. These three patients improved dramatically following the lavage [44].

It would appear from these case reports that NAC has a role in reducing lung inflammation and dislodging mucus secretion in mechanically ventilated patients.

### 4.6. Intravenous N-Acetylcysteine

IV NAC does not appear to be effective based on a placebo-controlled randomized study involving 38 long-term ventilated patients of a surgical intensive unit. Treatment with 3 g of IV NAC for 5 days did not show clinically relevant differences in their tracheobronchial mucus when compared to placebo. Additionally, there were no significant differences in their reduced glutathione levels in the plasma or in the bronchoalveolar fluid which suggested that there was no antioxidant benefit of NAC over placebo [45].

### 4.7. Mucoid Impaction—What Is It and Does N-Acetylcysteine Help?

Mucoid impaction, also referred to as mucus plugging or bronchocoele, is a term used to describe a clinical syndrome characterized by inspissated mucus plugs in the second-order bronchi [46]. Patients with mucus impaction may be asymptomatic or present with cough, chest pain, dyspnoea or expectoration of mucus plugs. It more frequently occurs in patients with obstructive airway disease due to either inflammatory or malignant conditions who live in warm geographic areas with low humidity. Mucoid impaction may sometimes result from non-obstructive causes which eventually obstruct the bronchi as well. The sequelae of mucoid impaction include distal pneumonitis, abscess formation, bronchiectasis and asymptomatic nodules. The mucus may affect the large airways and may be seen on chest radiographs as tubular or branching opacities. The finger-in-glove sign describes the opacities which typically radiate from the hilum towards the periphery of the lung though ovoid opacities are common.

A chest CT scan is usually the next modality of evaluation. A neoplastic cause should be considered in the presence of hilar adenopathy and endobronchial or perihilar masses while diffuse or multifocal bronchiectasis may suggest an underlying immune deficiency or systemic disease [47]. Central bronchiectasis and high-attenuation mucus (HAM) suggests ABPA [47]. Bronchoscopy may be indicated for further diagnostic evaluation or for treatment [48]. NAC is reported to have been successfully used to dissolve large long-standing plugs, thereby circumventing the need for surgical intervention such as lobectomy [49]. NAC has been reported to effectively reduce the size of large mucus plugs, thereby allowing for much smaller surgical resections at an elective interval where indicated [47].

### 4.8. Allergic Bronchopulmonary Aspergillosis and Hyperattenuated Allergic Mucin

**Figure 2:**
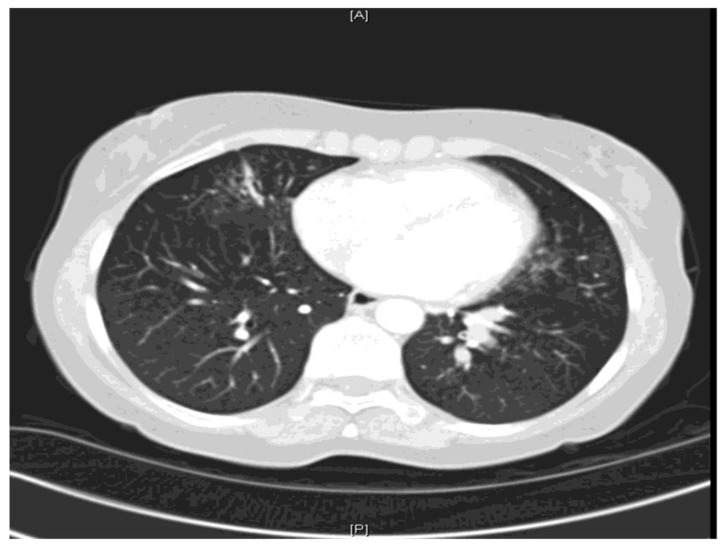
CT thorax showing extensive mucus plugging within the left lower lobe.

**Figure 3:**
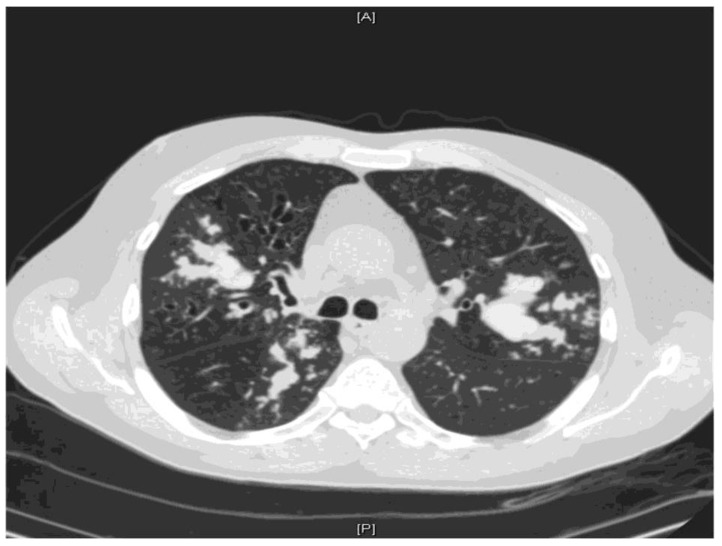
CT thorax showing severe multilobar bronchiectasis with accumulation of high-attenuation mucus forming bronchoceles predominantly within the right upper lobe, the right lower lobe and the left upper lobe.

ABPA is an immunologic disorder secondary to antigens released by *Aspergillus fumigatus* and occasionally related species. It is a hypersensitivity disorder which occurs in patients with either asthma or cystic fibrosis and presents with poorly controlled asthma, expectoration of mucus plugs occurs and features of co-existing bacterial infection are haemoptysis, weight loss and fever [50]. Excess mucus secretion with HAM is one of the most characteristic features of ABPA [51,52,53] and may result in mucoid impaction (Figure 2 and Figure 3).

HAM plugging it thought to be due to either the presence of calcium salts, iron and manganese [22] or desiccated mucus. Hemosiderin occurring within inspissated mucin was initially thought to account for the increased signal intensity but this was disputed by Zinreich and colleagues, who were unable to identify increased hemosiderin within typical allergic fungal mucin [23].

On a high-resolution computed tomography (HRCT) scan, the attenuation of mucoid impaction in many patients is higher than that of paraspinal skeletal muscle at CT Hounsfield values, 70–170 [24,25,26]. However, in some patients, mucoid impaction has a similar or lower attenuation than that of paraspinal skeletal muscle (CT Hounsfield values, 10–40) [25,27,28]. The presence of HAM has clinical implications as it been associated with recurrent relapses [23]. Unlike bronchiectasis [54], HAM has been reported to be marker of initial disease severity. It has been hypothesized that patients with HAM have specific genetic alterations which predispose them to developing HAM and more severe inflammation with poorer outcomes [28], but this is yet to be substantiated. There are no data on the use of NAC on HAM.

## 5. Conclusions

The management of unresponsive bronchial obstruction from inspissated mucus poses a serious challenge for both patients and clinicians alike. There are several mucoactive substances that act to increase the ability to expectorate sputum or to decrease mucus hypersecretion, and these have been used in several pulmonary diseases with variable success. To the best of our knowledge, this is the first report describing the use of a mucoactive substance in the management of persons with aspergillus lung disease. From the foregoing, it appears that NAC has modest activity in clearance of viscid bronchial mucus secretions in patients with aspergillus lung disease and should be considered for use in such settings.

## Figures and Tables

**Table 1:** Characteristics of patients in the National Aspergillosis Centre, Manchester, treated with 20% NAC at a dose of 4mL twice a day.

Sex	Age	Diagnosis	Co-Morbidities	Duration of NAC Use	Outcome	FEV1 Pre/Post (Liters)	Any Other Relevant Clinical Information?
Female	50	CPA, ABPA	Asthma, bronchiectasis, adrenal insufficiency, and previous left upper lobectomy for bronchiectasis	14 days	Discontinued. No change in symptoms	1.67/1.53	Trialed in order to try and avoid bronchoscopy. She was already receiving daily physiotherapy after 8 mL of 7% saline via nebulizer. The challenge with NAC was followed by intermittent positive pressure breathing therapy, 8 mL 7% saline and active cycle of the breathing technique
Male	63	ABPA	Asthma, coeliac disease, sinusitis, hypertension vitamin D deficiency, liver cysts, and hyperaldosteronism	1	Failed initial challenge due to increased breathlessness. However, he was able to expectorate freely and provided a sputum sample which he hadn’t managed to do previously	2.25/1.96	Had not taken his usual dose of Fostair/salbutamol that morning. He was noted to be wheezy pre-challenge. Post NAC challenge, he was given 2 puffs of salbutamol via a metered dose inhaler.
Female	59	ABPA	Asthma, vocal cord dysfunction, bronchiectasis, and tracheal stenosis	Long term since 2/10/2014	Discontinued. No change in symptoms	Not performed-tracheostomy patient. No change on auscultation/pulse oximetry (98%)/heart rate	Prior to challenge that had already self-suctioned 6 times that morning. Already nebulizing budesonide, salbutamol, 7% saline and colistin
Male	47	ABPA	Right upper lobectomy	1 week	Discontinued. No change in symptoms	4.15/4.10	Never tried 7% saline via a nebulizer
Female	57	*Aspergillus terreus* and *Trichoderma* bronchitis	Hypothyroidism, migraine, and bronchiectasis	8 months. A clinical decision was made to stop NAC as she felt much better post bronchoscopy	Well tolerated, increased expectoration of phlegm	1.99/1.89	Already on 7% saline via nebulizer. Underwent bronchoscopy with lavage 8 months after commencing NAC
Female	83	ABPA Recurrent left lower lobe collapse	Hypertension, osteoarthritis, glaucoma, and nasal disease of uncertain aetiology	7 months	Tolerated NAC. Eventually discontinued as she derived greater benefit from 7% saline nebs		NAC appeared to aggravate her cough. No further lobar collapse recorded

ABPA = allergic bronchopulmonary aspergillosis; CPA = chronic pulmonary aspergillosis; NAC = *N*-acetylcysteine.

**Table 2:** Mucoactive substances in clinical use.

Drug	Device	Indication	Proposed Mechanism of Action	Notes
*Expectorants*				
Hypertonic saline 7%	Nebulizer	Cystic fibrosis, and bronchiectasis	Increases the amount of sodium and chloride in airway surface liquid, thereby increasing the osmotic gradient and rehydrating the mucus layer [5,7]	Improves lung function and quality of life in bronchiectasis [8]. Should not be given via a vibrating mesh nebulizer. Improves mucus clearance, airflow, and reduces rates of exacerbation among patients with cystic fibrosis [4,9].
*Classical mucolytics*				
NAC (Mucomyst^®^)	Nebulizer	ABPA	Severs disulfide bonds that link mucin monomers to polymers, and solubilizes sputum antioxidant and anti-inflammatory	No evidence for use in any lung disease.
S-carboxymethylcysteine (carbocysteine)	Oral	COPD, and cystic fibrosis	Increases concentrations of sialomucins and reduces that of fucomucins, acts as a free radical scavenger [10], and has antioxidant and anti-inflammatory properties	Reduces measured sputum viscosity [11,12].
Dry powder mannitol (Bronchitol^®^)	Dry powder inhaler	Cystic fibrosis, bronchiectasis, and COPD	Increases mucus secretion	Nonabsorbable. Associated with bronchoconstriction and cough when used in children with cystic fibrosis.
*Peptide mucolytics*				
Dornase alfa (Pulmozyme^®^)	Nebulizer	Cystic fibrosis	Hydrolyzes DNA polymer and reduces DNA length	Hydrolyzes DNA, improves lung function, and decreases the frequency of exacerbation [13,14].
*Nondestructive mucolytics*				
Unfractionated heparin (UFH)	Nebulizer	COPD, and cystic fibrosis	Modifies ionic interactions and the intermolecular hydrogen bonds between mucin molecules, and untangles the charged oligosaccharide side chains of mucin	UFH reduces the elasticity and yield stress in the samples from cystic fibrosis patients [15].
Low molecular weight dextran (DCF 987)	Nebulizer	COPD	Disrupts the polyionic oligosaccharide mucin network and increases secretion hydration	Proven lung safety in animal studies [16,17].

ABPA = allergic bronchopulmonary aspergillosis; COPD = chronic obstructive pulmonary disease; NAC = *N*-acetylcysteine.

**Table 3:** NAC products on the market.

Brand	Preparation	Administration	Side Effects
Mucomyst^®^ by Bristol-Myers Squibb	Sterile unpreserved solutions (not for injection) of 20% (Mucomyst-20) or 10% (Mucomyst-10) acetylcysteine, with edetate disodium in purified water. Sodium hydroxide is added to adjust pH to 7.	Nebulization using face mask, mouth piece, tracheostomy, tent or croupette, direct introduction into a segment of the bronchopulmonary tree via a plastic catheter, and can also be given via a percutaneous intratracheal catheter and during bronchoscopy.	Stomatitis, nausea, vomiting, fever, rhinorrhea, drowsiness, clamminess, and bronchoconstriction. Acquired sensitization to NAC may rarely occur.
Fluimucil by Zambon	Capsules 200 mg, effervescent tablets 600 mg, paediatric sachet 100 mg, granules 200 mg, dry syrup 100 mg/5 mL, and injection suspension (ampoule) 300 mg/3 mL	Oral and aerosol administration	Capsule/granule/dry syrup: heartburn, nausea, vomiting, diarrhoea, stomatitis, dizziness, tinnitus, allergic and reduced blood pressure. IV: hypersensitivity reactions, rhinorrhea, stomatitis, nausea and vomiting.
Acetadote^®^ injection by Cumberland Pharmaceuticals Inc. Nashville, TN 37203	20% solution in 30 mL (200 mg/mL) single-dose glass vials, preservative-free for IV administration.	For IV administration	Anaphylactoid reaction due to pyrogens, flushing, oedema, urticaria, pruritus, nausea, pharyngitis, rhinorrhea
Cetylev^®^ tablets by Alpex Pharma SA	Effervescent tablets 500 mg or 2.5 grams of NAC.	Oral administration	Nausea, vomiting, other gastrointestinal symptoms, and rash with or without fever.
